# 

**Published:** 1887-06

**Authors:** 


					﻿/VOL Vi/
JUNE, 1887.
NO. 6.
THE
'Homeopathic ph^ici<
A MONTHLY JOURNAL OF MEDICAL SCIENCE.
“ He is a freeman whom truth makes free, and all are slaves beside.”
“Seek the Truth: come whence it may, cost what it will.”
EDITED BY
Edmund j. lee, m. d.,
AND
WALTER M. JAMES, M. D.
PHILADELPHIA :
No. 11B3 SPRUCE STREET.
JTSJW ■3rO2R»,2K»
A. L. CHATTERTON & COMPANY, Publisher’s Agents.;
LONDON'S
ALFRED HEATH & CO., Sole Agents fob Great Britain,
114 Ebury Street, Eaton Square.
Yearly Subscription, $9.50, invariably in advance. Single numbers, 95 cts.
Entered at the Philadelphia Poet-Office as Second-Claae Mail Matter.
				

